# Osmotic pump with potential for bone lengthening distracts continuously *in vitro* and *in vivo*

**DOI:** 10.1371/journal.pone.0291335

**Published:** 2023-09-14

**Authors:** Sebastian Lippross, Heiko M. Lorenz, Lena Braunschweig, Andreas Heede, Robin Büscher, Marlon Siegel, Gerhard Schultheiß, Sarah Vieten, Annette Lüthje, Andrea Matzen, Katja A. Lüders, Katharina Jäckle, Konstantinos Tsaknakis, Anna K. Hell

**Affiliations:** 1 Department of Orthopaedics and Trauma Surgery, University Medical Center Schleswig-Holstein, Kiel, Germany; 2 Pediatric Orthopaedics, Department of Trauma Surgery, Orthopaedics and Plastic Surgery, University Medical Center Goettingen, Goettingen, Germany; 3 Stryker Trauma GmbH, Schoenkirchen, Germany; 4 Department of Animal Welfare, CAU Kiel, Kiel, Germany; 5 Department of Internal Medicine III, UKSH University Hospital of Schleswig-Holstein Kiel Campus, Kiel, Germany; 6 Department of Trauma Surgery, Orthopaedics and Plastic Surgery, University Medical Center Goettingen, Goettingen, Germany; University Hospital Zurich, SWITZERLAND

## Abstract

**Background:**

In pediatric orthopedics, long bone lengthening procedures are routinely performed using manual, motorized or magnetically controlled implants. This study aims to prove expansion of a newly designed osmotic pump prior to long bone lengthening in living organisms and to rule out any complications related to *in vivo* conditions, such as congestion of the semipermeable membrane, local infection, or lack of water to drive the osmotic pump, as well as to compare *in vivo* and *in vitro* expansion data.

**Methods:**

Osmotic pumps, which were designed to distract a plate osteosynthesis, were inserted in the dorsal paraspinal musculature of four piglets. To compare the performance of the pumps in *in vivo* and *in vitro* conditions, another set of pumps was submerged in physiologic saline solution at different temperatures. The lengthening progress was measured radiographically and sonographically in the study animals.

**Results:**

Both, *in vitro* and *in vivo* tested osmotic pumps started distraction after an intended rest phase of four days and distracted evenly over the following twelve days. No complications, clogging or damages occurred. However, we observed a temperature dependency of the distraction rate ranging from 0.98 mm/day at 39°C to 1.10 mm/day at 42°C. With a second setup, we confirmed that the distraction rate differed by 72% within a measured temperature interval of 14° C.

**Conclusions:**

The data presented here confirm that the novel osmotic pump showed comparable lengthening characteristics *in vivo* and *in vitro*. No complications, such as congestion of the semipermeable membrane, local infection, or lack of water to drive the osmotic pump were observed. Thus, osmotic pumps may have great potential in future applications such as long bone lengthening procedures, where continuous distraction probably provides a better bone quality than intermittent lengthening procedures. The fact that one pump failed to elongate in each condition, highlights the importance of technical improvement, but also demonstrates that this was not due to different circumstances within the *in vivo* or *in vitro* condition.

## Introduction

Limb lengthening is a fast-growing field of orthopedic surgery for the treatment of congenital or acquired limb length discrepancies and/or for aesthetic reasons [1]. The majority of current and newly developed treatment methods are related to the Ilizarov’s technique based on the tension-stress effect, which states that slow and gradual distraction stimulates new bone formation and soft tissue regeneration [2]. However, limb lengthening procedures by the Ilizarov apparatus or similar external fixateur devices have a high complication rate with up to 84% [1, 3, 4] in addition to considerable cosmetic and functional impairment [5, 6]. The most common complications include pin site infection, breakage of pins, axial malalignment and joint subluxation or dislocation [1]. Intramedullary nails are an alternative treatment method, but their application is limited to adolescents and adults as they require a certain diameter of the tubular bone and potentially damage the growth plates in younger individuals [3]. The increasing demand for an applicable method for lower extremity lengthening in young children, which bears less complications, is reliant and more easy to use, led to the development of a plate-assisted lengthening technique [7, 8].

For lengthening implants, mechanical, electromechanical, electromagnetic and osmotic drives can be considered. While magnetically controlled implants became increasingly popular in the last decade [9–13] several complications, such as metallosis, leakage, corrosion and pin breakage, have been reported, leading to a temporal withdrawal of some of these implants from the market [14]. For externally controlled motorized intramedullary bone lengthening nails the overall risk of complications was one complication for every three lengthened segments [10]. Frost et al. [15] found a 53% complication rate (n = 257 patients) analyzing Fitbone^®^ and Precice^®^ results. Miniature osmotic actuators have been investigated for maxillofacial distraction [16] but to our knowledge, no osmotic-driven devices have been proposed and investigated for the use in limb lengthening yet.

Osmosis is defined as the net movement of water across a selectively permeable membrane, a process that is driven by a concentration gradient. Due to the continuous mode of action and the independence from external control, osmotic pumps have great potential for several applications in medicine, such as drug administration and bone distraction. Animal studies on bone healing in the mandible have shown that continuous osteodistraction leads to faster bone healing than noncontinuous lengthening [17]. Also, subcutaneously implanted, miniature osmotic pumps have already been used as drug pumps in experimental animals to achieve continuous long-term drug delivery [18]. However, those pumps were intended for systemic administration of drugs, hormones and other test agents in animal models only and studies of other applications of osmotic pumps are rare.

In this study, we evaluated the feasibility and efficacy of an osmosis driven pump that was designed to actuate plate osteosynthesis for long bone lengthening, using piglets as an experimental *in vivo* system.

## Methods

This study was performed in accordance with the German law for animal protection. All experiments were evaluated by the Local Institutional Animal Care and Research Advisory committee and permitted by the local authorities (Ministry of Energy, Agriculture, the Environment, Nature and Digitalization, MELUND; approval number: V 242–76992/2021 (1-1/22)). Severity assessment performed by the facility staff was requested by authorities.

Piglets were chosen as study model, since their size and anatomy is similar enough to humans, so that the osmotic pump could be tested in the space between the muscles, similar to the final destination to the pump when applied as a bone lengthening device. The implantation space between the back muscles was chosen for its easy access and its minor impact on the piglets’ wellbeing. After study approval, four immature six weeks old, female piglets (domestic pig breed) with an average weight of 18.7 kg ± 1.3 kg were transferred from the breeding farm Sye GbR (Prasdorf, Germany) to the Department of Animal Welfare of Kiel University, Germany. Upon arrival, an unaffected health status was verified by a veterinarian. The piglets were housed as a flock in an indoor enclosure and were fed feed pellets with water provided ad libitum. During the adjustment time of six days before surgery, animals were familiarized with the surroundings and the ultrasound investigations on the back. Twelve hours prior to surgery food was discontinued.

**Fig 1:**
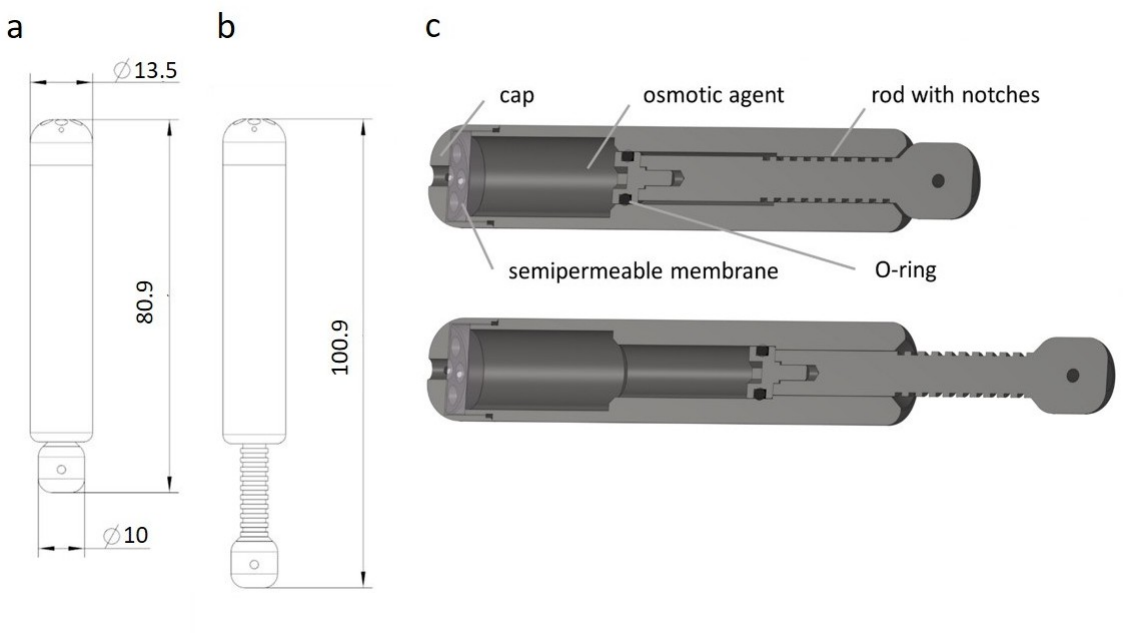
Schematic image of the osmotic pump in the original **(a)** and fully distracted **(b)** state. 3D models **(c)** of the osmotic pump in original **(top)** and fully distracted **(bottom)** state. The osmotic pump consists of a cylinder with a removable cap, a semipermeable membrane, an inner core filled with the osmotic agent, and a notched rod. The values given indicate length and diameter in cm.

A cylindrical osmotic pump was designed using a semipermeable membrane and high-concentrated saline to enable the osmotic process (Fig 1). The inner core absorbs water through a membrane and defined holes in the cap and thereby expands its volume, while pushing out a rod continuously. The pump was designed to have an initial rest phase of four to five days after implantation to allow cell proliferation after the osteotomy, and subsequent lengthening of approximately 1 mm per day. For ultrasound monitoring of the implant expansion, the inner rod had 0.5 mm deep notches with a specific width of 1 mm and a distance of 1 mm to each other. The maximum distraction length of the rod is 20 mm.

**Fig 2:**
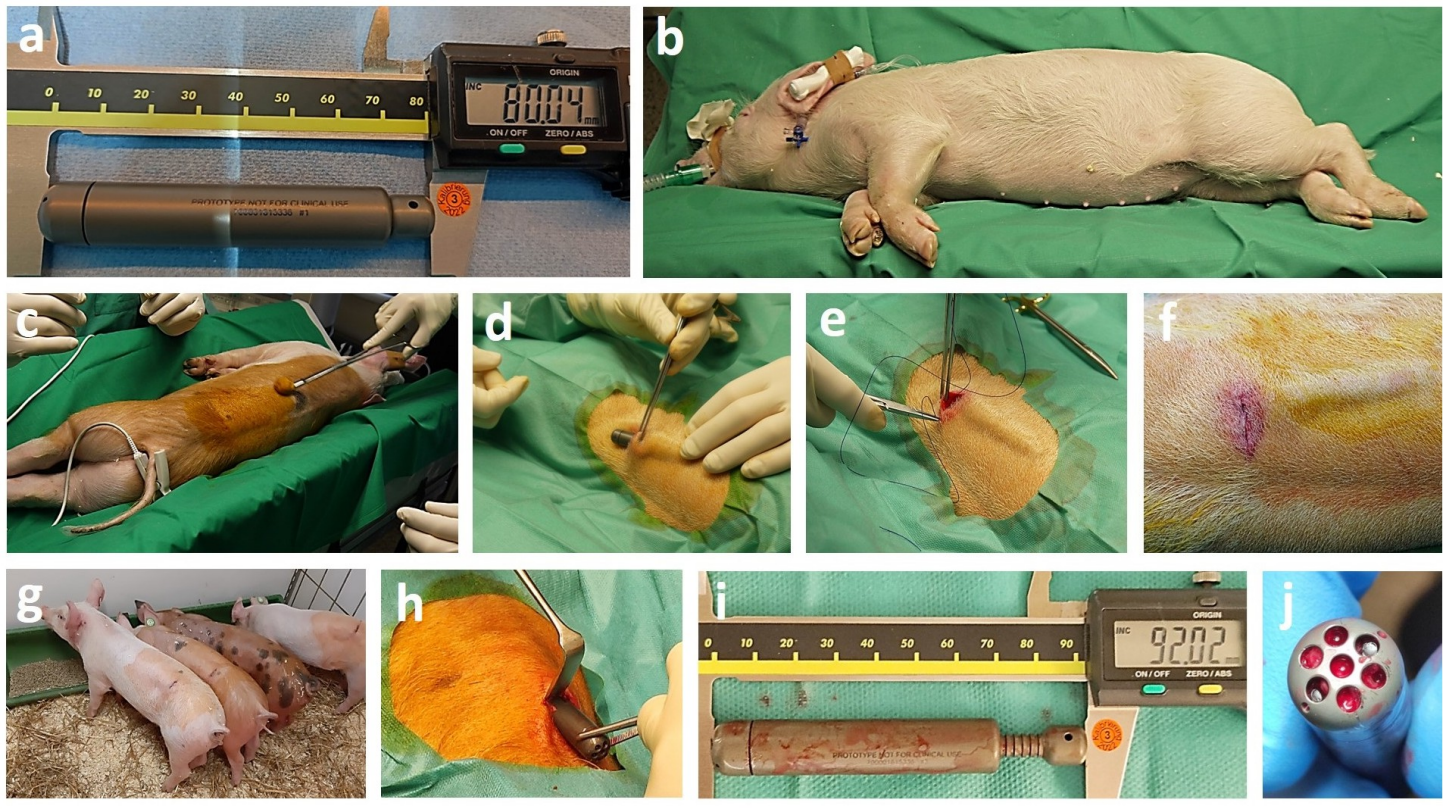
**(a)** Unwrapped osmotic pump directly before implantation measuring 8.0 cm. **(b)** Piglet in narcotic state and lateral position before surgery. **(c)** Multiple disinfection of the back, where the implantation site is planned. **(d)** Insertion of the pump between the back muscle (*longissimus dorsi*) and the muscle fascia. **(e)** Layered skin closure. **(f)** Back of the piglet with pump implanted. **(g)** All four piglets with the pump implanted some hours after surgery. **(h)** Explantation of the pump after 16 days. **(i)** Retrieved pump measured 92. **(j)** Inflow holes to the osmotic membrane after retrieval.

For surgical insertion of the implants into the piglets, anesthesia was performed using intraperitoneal injection with ketamin/xylazin and atropinsulfat as well as propofol (propofol Lipuro 2%, 15 mg/kg) and isoflurane (1.5%). After animal positioning, shaving, multiple disinfections and sterile draping, a small incision on the back of the piglets was performed with preparation of the back muscles (*musculus longissimus*) for implant insertion. The gamma ray-sterilized, 8 cm long osmotic pump was subfascially inserted and the wound was closed in layers by using resorbable sutures (Fig 2a–2f). Radiographs were taken to ascertain the position and state of the pump directly after the implantation. Total anesthesia and surgical time were 60 minutes for each piglet.

For pain management the animals received intramuscular meloxicam 5% (0.4 mg/kg) before surgery and orally applied Carprofen (2 mg/kg) for three days post surgery. Furthermore, prophylactic intramuscular amoxicillin clavulanic acid (8.75 mg/kg) was applied for three days.

Implantation of the osmotic pump in the dorsal musculature of piglets was successful in all four cases. The piglets were alert and active after waking up from anesthesia. During the entire follow-up period, no medical complications could be observed. After surgery, the piglets were kept as a flock in the indoor enclosure and were observed daily using a score sheet, to document the piglets’ activity, behavior, food and water uptake, appearance, breathing, temperature and wound healing including symptoms of infection. Every morning, ultrasound on the back of the piglets was performed with a mobile ultrasound scanner (Examion, Germany, linear transducer). Due to a distance indicator in terms of 1 mm broad notches, ultrasound imaging allowed daily estimation of distraction length of each of the four inserted pumps. Sixteen days after implantation, the pump was explanted following the above-mentioned anesthesia and surgical procedure. For comparison, radiographs were obtained. The length of the osmotic pump was measured directly after retrieval (Fig 2i). Additionally, the pump was checked for residues of tissue, obvious damages and clogging of the inflow holes (Fig 2h–2j). The piglets recovered from surgery, thus sacrifice of the animals was not necessary.

**Fig 3:**
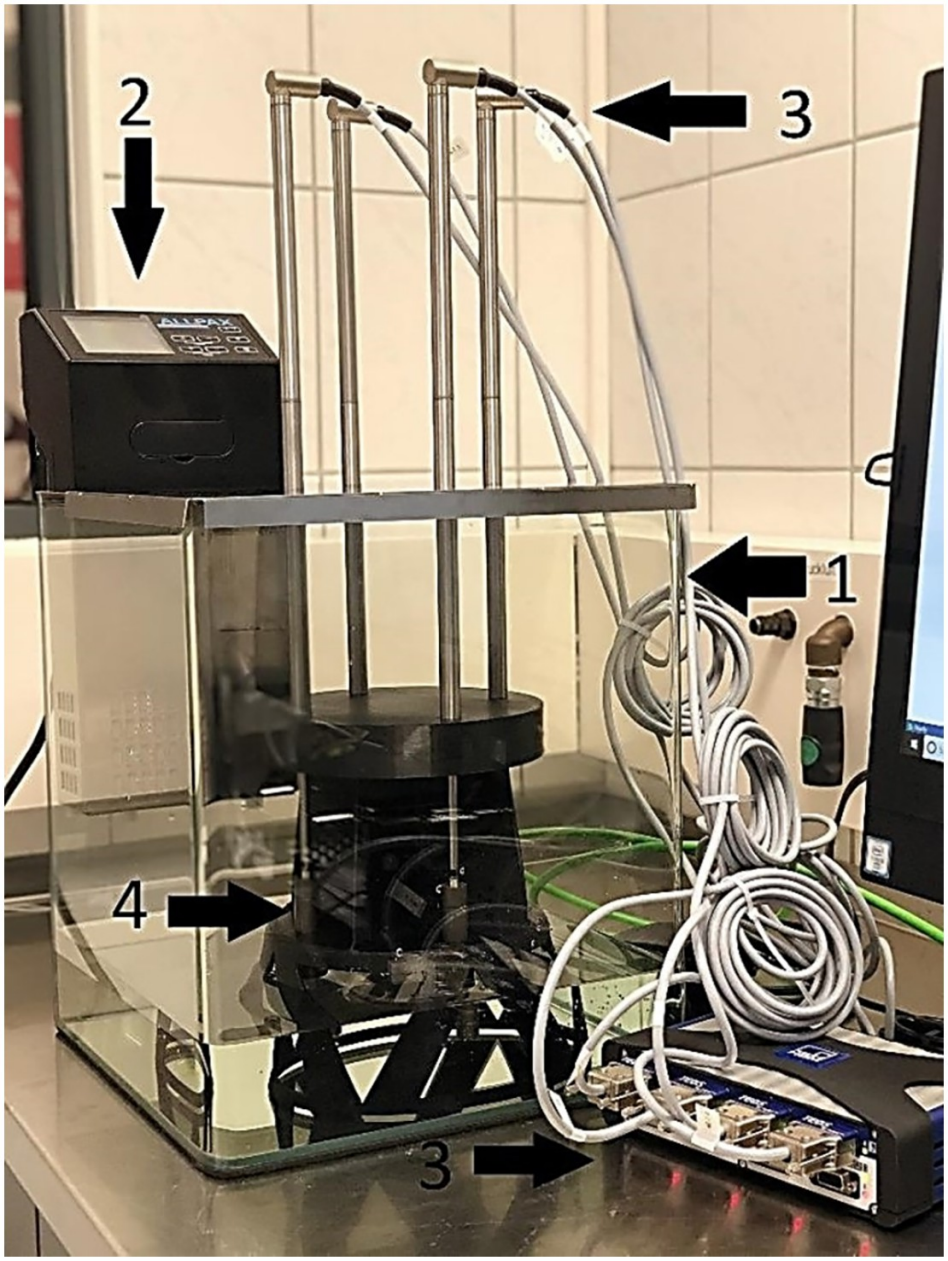
Setup to test the osmotic pumps at *in vitro* conditions. **1:** Water bath filled with 0.9% saline solution. **2:** Heating for the desired water temperature. **3:** Displacement transducers to measure lengthening of the osmotic pumps. **4:** Rack with osmotic pumps.

During the period of *in vivo* experiments, simultaneous testing of the implants was performed *in vitro*. On the day of surgery, four pumps of the same manufacturing lot were positioned in a water bath filled with 42 °C saline solution of 0.9% NaCl under laboratory conditions (Fig 3). Displacement transducers sent the data to a computer where the HBM catman Acquisition Software (Hottinger Brüel & Kjær, Virum, Denmark) tracked them in real-time. Because 42 °C was accidentally set, three additional pumps were tested at 39 °C in the same setup, which corresponds to the estimated temperature in the back muscle of the pig [19].

To calculate the daily distraction rate of the osmotic pumps, the distraction length (i.e. length-difference between experimental start and end) was divided by the time the pumps actually distracted without the initial four-day rest phase (i.e. 12 out of 16 days) for *in vivo* and *in vitro* pumps. Additionally, the exact length and distraction rate of the *in vitro* pumps was automatically measured daily in the setup.

After noting the temperature dependency of the osmotic pump distraction rate, an additional *in vitro* testing was performed with a new setup to evaluate and validate the temperature dependency of the osmotic pump in general. The temperature of the saline solution was constantly measured and adjusted every 50 seconds to the following values: 32 °C (average in real-time measurements 32.17 °C, range 31.13–33.38 °C), 38.5 °C (average in real-time measurements 38.63 °C, range 37.44–39.85 °C), and 46 °C (average in real-time measurements 46.33 °C, range 45.13–47.47 °C). Furthermore, the pumps were prepared to start distraction without a rest phase, thus absolute distraction rates cannot be directly compared with the results from the previous experiments, but within this setup.

## Results

**Fig 4:**
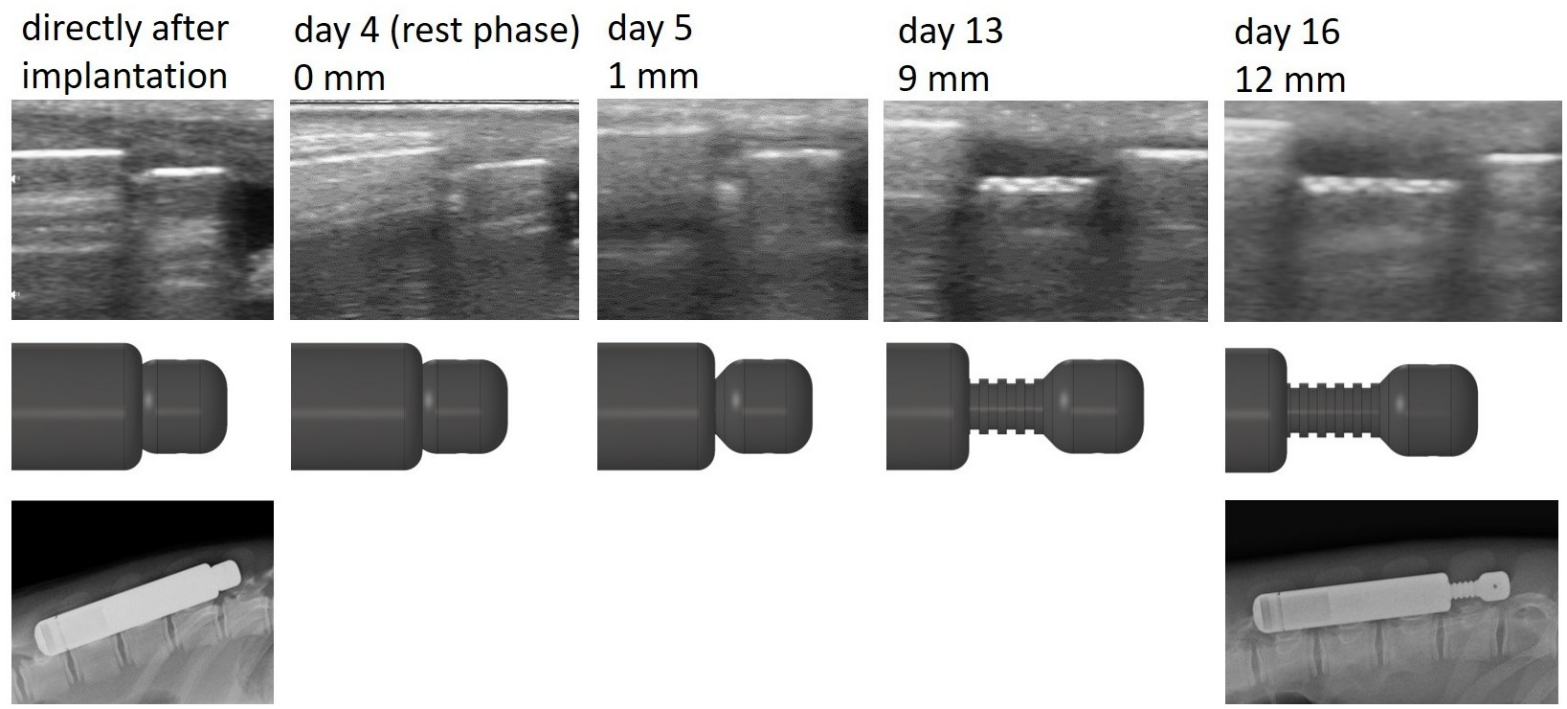
Series of ultrasound scans **(upper row)** and distraction in scheme **(mid row)** directly after implantation, on day 4 (last day of rest phase), day 5 (first day of elongation), day 8, day 13 and right before explantation on day 16. Notches with 1 mm width served as a distance indicator in the ultrasound scans. X-ray imaging **(bottom row)** was performed directly after implantation and before retrieval, both with anesthesia.

Within the score-sheet documentation of the well-being after surgery all 4 piglets achieved the lowest score of stress level (null points) each day. Radiographs and ultrasonic images taken directly after implantation surgery showed the desired position in the dorsal musculature of all four piglets (Fig 4).

Subsequent daily ultrasound scanning *in vivo* revealed that the osmotic pumps started elongation after the desired rest phase of four days after implantation (Fig 4), which was analogous to the *in vitro* pumps inserted in 42 °C saline solution. Even more importantly, the osmotic pumps implanted in piglets reached also comparable lengths of extension as observed with the *in vitro* pumps (Table 1). In both conditions, one of the four pumps failed due to a manufacturing defect, i.e. leakage of the saline solution inside the pump through the joint /O-ring (pumps number 2 and 5; Table 1). The reason for this failure was a small burr on the edge of the O-ring groove that damaged the ring itself. Thorough investigation of the explanted pumps at the end of the animal test showed that the three functioning *in vivo* pumps had extended by 11.77 (±0.39) mm on average. Neither clogging of the inflow holes of the osmotic pump towards the semipermeable membrane nor tissue infection could be observed.

**Table 1:** Measured lengths of the osmotic pumps at the start and end of the experiment in mm.

		Measured length at experimental start (in mm)	Measured length at experimental end (in mm)	Distraction length (in mm)	Average of the functioning pumps of each group (in mm)
	**pump 1 (*in vivo*)**	80.04	91.36	11.32	**11.77 (±0.39)**
	** $\begin{array}{l} pump\;2\left(in\;vivo\right) \end{array}$ **	79.93	80.05	0.12	
	**pump 3 (*in vivo*)**	80.02	92.02	12.00	
	**pump 4 (*in vivo*)**	79.90	91.90	12.00	
$\begin{array}{l} 42{}^{\circ}\text{C} \end{array}$	** $\begin{array}{l} pump\;5\left(in\;vitro\right) \end{array}$ **	79.90	79.97	0.07	**13.14 (±0.40)**
42 °C	**pump 6 (*in vitro*)**	79.73	93.22	13.49	
42 °C	**pump 7 (*in vitro*)**	79.86	93.08	13.22	
42 °C	**pump 8 (*in vitro*)**	79.92	92.63	12.71	
39 °C	**pump 9 (in vitro)**	79.82	91.94	12.12	
39 °C	**pump 10 (in vitro)**	79.85	91.21	11.36	**11.74 (±0.54)**
$\begin{array}{l} 39{}^{\circ}\text{C} \end{array}$	** $\begin{array}{l} pump\;11\left(in\;vitro\right) \end{array}$ **	$\begin{array}{c} 79.90 \end{array}$	$\begin{array}{c} 89.94 \end{array}$	$\begin{array}{c} 10.04 \end{array}$	

For both, the *in vivo* and *in vitro* measurements at 42 °C, one pump failed (pump 2 and 5, values in grey and was not used for the average value). One *in vitro* pump at 39 °C water started to elongate later than planned after six instead four days (pump 11, values in grey and were not used to calculate the average value).

The functioning *in vitro* pumps at 42° showed an overall lengthening of 13.14 (±0.40) mm, which is slightly, but significantly different from the *in vivo* results (p = 0.013). As the cause of the different elongation, the suggestion was that this finding is due to the temperature difference *in vivo* ~39 ° C [19] to *in vitro* 42 ° C. Therefore, another *in vitro* test with 39 °C was repeated with a new set of three pumps. Two of these pumps started distraction after a rest phase of four days and showed a lengthening of 11.74 (±0.40) mm on average, which is not significantly different from *in vivo* results (p = 0.940). One of these pumps (number 11; Table 1) started after six days, which might indicate a technical defect.

**Fig 5:**
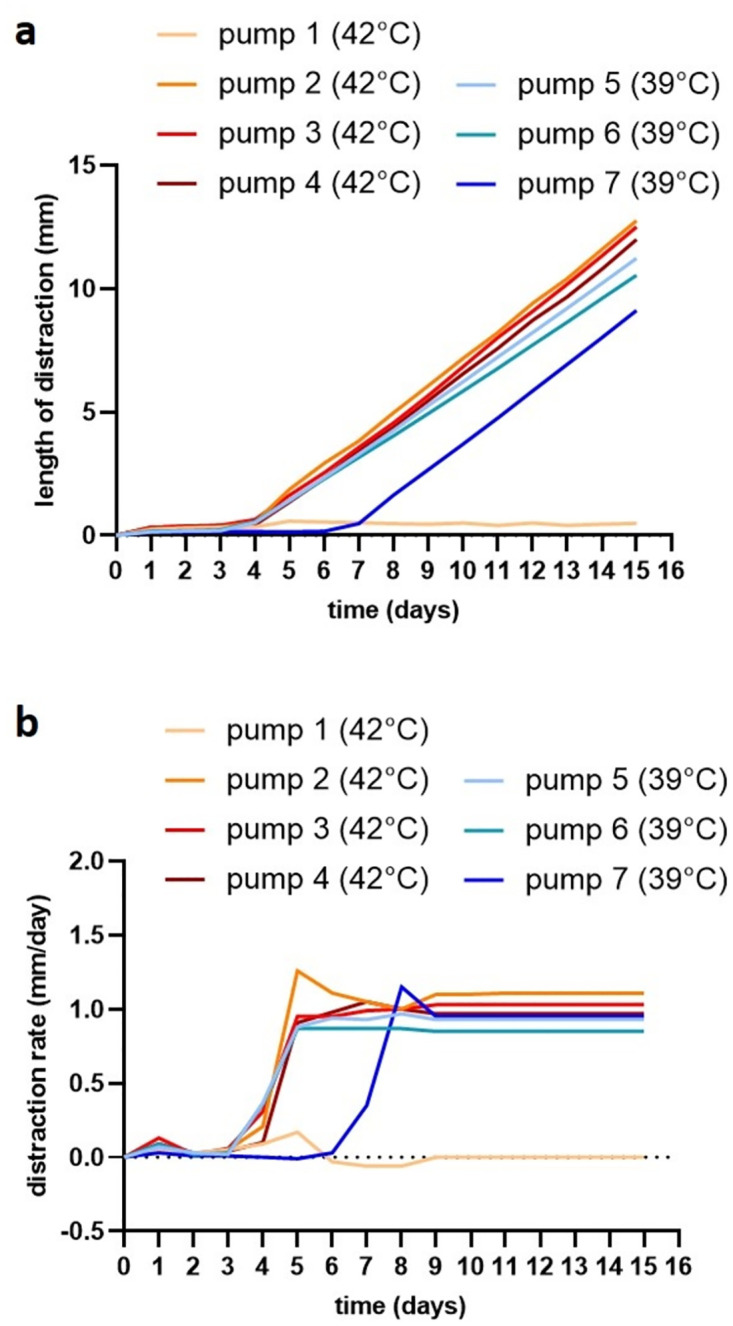
Distraction curves (a) and distraction rate (b) of the seven *in vitro* pumps. Four *in vitro* pumps (pumps 5–8) were started at 42 °C parallel to the *in vivo* pumps implanted into piglets. After the desired rest of four days after experimental start, continuous distraction was observed in three of four cases until the end of follow-up at day 16. Out of the three *in vitro* pumps started at 39 °C, two pumps (pump 9 and 10) started to distract after the intended rest phase of four days, whereas one pump (pump 11) started after six days.

The daily distraction rate, calculated based on the length the pumps gained and the time they distracted, was compared to the daily values measured under laboratory conditions (Table 2 and Fig 5). For the three functioning *in vivo* pumps a daily distraction rate of 0.98 (±0.03) mm/day was calculated, based on the observation that the pumps started to distract after a rest phase of four days in ultrasound examinations. For the three functioning *in vitro* pumps at 42 °C, which also started to distract after four days, the daily distraction rate was 1.10 (±0.03) mm/day, which was significantly different from the *in vivo* results (p = 0.013). At 39 °C, osmotic pump distraction did not differ *in vivo* and *in vitro* (p = 0.940; Table 2).

**Table 2:** Daily distraction rates in mm/day.

Temperature	Osmotic pump number	Daily distraction rate (in mm/day)	Average (in mm/day)
39 °C	**pump 1 (*in vivo*)**	0.94	
39 °C	**pump 3 (*in vivo*)**	1.00	**0.98 (±0.03**)
39 °C	**pump 4 (*in vivo*)**	1.00	
42 °C	**pump 6 (*in vitro*)**	1.12	
42 °C	**pump 7 (*in vitro*)**	1.10	**1.10 (±0.03)**
42 °C	**pump 8 (*in vitro*)**	1.06	
39 °C	**pump 9 (in vitro)**	1.01	
39 °C	**pump 10 (in vitro)**	0.95	**0.98 (±0.06)**

The daily distraction rates of all functioning *in vivo* and *in vitro* pumps were calculated by dividing the elongated distance of the pump by the number of days the pumps distracted (time of experiment without the initial rest phase, i.e. 16–4 = 12 days).

The daily measurements of distraction rates of the *in vitro* pumps revealed, that it took the pumps only one day to reach the desired speed with a distraction rate of 1.04 (±0.19) mm/day. The length of distraction increased very evenly (Fig 5a), with a constant daily distraction rate (Fig 5b). From day 9 after the start of the experiment onwards, the elongation rate within each pump even remained constant until the second number behind the comma with 1.03 (±0.07) mm/day, indicating a very consistent distraction of the osmotic pump over time. There was no significant difference between the calculated and the measured distraction rates for the *in vitro* pumps run at 42 °C (p = 0.217), showing that the calculation based on the distracted distance over time after the rest phase leads to valid values of the distraction rate. The same holds true for the *in vitro* pumps run at 39 °C with a calculated distraction rate of 0.98 (±0.06) mm/day and a measured distraction rate of 0.89 (±0.06) mm/day (p = 0.226).

Comparing the distraction rates of the pumps under the conditions of the additional setup confirmed that temperature had indeed an impact on the velocity of the lengthening process. Average distraction rates of the three pumps were 0.527 (±0.025) mm/day at 32.2 °C, 0.826 (±0.018) mm/day at 38.6 °C and 0.907 (±0.037) mm/day at 46.3 °C. Thus, within the measured temperature range of 14 °C, the distraction rate increased by 72% from 0.527 mm/day to 0.907 mm/day. Taking 38.8 °C as the physiological temperature in animals [19], the distraction rate was affected more by lower temperatures (decrease by 64% comparing 38.6 °C with 32.2 °C) than by higher temperatures (increase by 10% comparing 38.6 °C with 46.3 °C).

## Discussion

This study evaluated the primary performance and efficacy of an osmotic pump designed to drive continuous osteodistraction in living organisms by comparing *in vivo* to *in vitro* results. Out of eleven tested osmotic pumps (n = 4 *in vivo*, n = 7 *in vitro*), three did not function as planned, while the remaining eight pumps started elongation after the intended rest phase of four days both *in vitro* and *in vivo*. Possible *in vivo* complications such as congestion of the semipermeable membrane, local infection of the surrounding tissue or lack of water in the environment to drive the osmotic pump did not occur in any case. The only complication observed was the malfunctioning of three out of eleven pumps, which was due to a technical defect of the pumps, i.e. a small burr on the edge of the O-ring groove that damaged the ring itself. A small chamfer on this edge will eliminate this problem. Pump 11 (*in vitro*) was operated under the same conditions as the pumps 9 and 10 (*in vitro*) but started running two days later. No technical defect could be detected visually, but a jamming of the mechanical components at the beginning of the elongation seems likely without clear evidence. In 2023, Frost et al. [15] found 32% device related complications per lengthening segment analyzing the Fitbone^®^ and Precice^®^ implants, 22 (per 314 segments) directly related to the distraction mechanism. Their device related complication rate is in line with the presented data of this study. However, thorough analysis of the osmotic device will most likely eliminate technical problems in the future.

In the animals, routine ultrasound examinations every 24 hours revealed the start of osmotic pumps after an intended rest phase of four days, which was equal to the *in vitro* data. The daily distraction rate *in vivo* was 0.98 mm/day. The calculated *in vitro* elongation rates were 1.10 mm/day at 42°C and 0.98 mm/day at 39°C, with the latter temperature corresponding to the physiological temperature in pigs [19]. In a clinical setting, this temperature dependent difference in the daily elongation will most likely not affect the callus formation but may lead to an earlier end of distraction when the intended elongation is reached.

There is a strong correlation between the distraction rate of the pump and the environmental temperature. Increased kinetic energy of particles at higher temperature and proportional relationship between temperature and the diffusion coefficient are described by physical laws. Xie et al. [20] observed that the infusion rate of an osmotic pump was increased by 12% at a temperature change of 4 °C. This value is consistent with our data, which show an increase of the lengthening rate by 11.6% of the *in vitro* osmotic pump at 42 °C when compared to the *in vivo* pumps at estimated 39 °C environment. Investigations of the temperature effect on osmotic pumps *in vitro* revealed that higher temperatures led to increased distraction rates whereas lower temperatures decelerated the pump. However, the temperature effect was more obvious for the temperature range below the physiological scale, while increasing the temperature above 38 °C had considerably lower impact. The same result was obtained by Wang et al. who observed a more pronounced osmosis rate from 25 °C to 35 °C, rather than from 35 °C to 45 °C [21]. The reason for this difference is that the decrease in relative viscosity of the draw solution of the osmotic system, i.e. saline solution, is more apparent with the temperature increase form 25 °C to 35 °C as compared to the temperature shift from 35 °C to 45 °C [21]. Decreased viscosity of the draw solution increases the diffusion coefficient and thus enhances mass transfer. Considering these findings, application of osmotic pumps in clinical practice presupposes a thermostatic environment. However, a variance of 4 °C is possible in the human body and this fact should be taken into consideration when adjusting the maximal efficacy of osmotic pumps, i.e. the maximum limit of drug delivery or maximal desired distraction length per day. As the presented pump was designed to drive osmotic osteodistraction for the lengthening of long bones, the temperature effect has to be thoroughly considered. The estimated temperature in human body is approximately 37 °C. However, the temperature depends on a variety of factors, including the activity level, clothing and environmental conditions, which might have a strong impact on the performance of the implanted pump as well as viral infections, which are commonly seen in children. Additional factors that may influence the elongation rate may be the salt content, the amino acids or proteins in the body fluid compared to the *in vitro* salt solution, as well as forces by muscles and body weight. As the presented data show, there is a temperature dependent effect of the osmotic driven distraction, which is more severe outside the physiological temperature range. Therefore, the clinical impact will be neglectable on the daily basis and only has to be taken into account when calculating the end of the distraction phase.

Limitations of this study include a relatively small sample size number of initially n = 4 (four pumps implanted *in vivo* in piglets and four pumps tested *in vitro*.) Malfunction due to technical defects further reduced the sample numbers. Fortunately, this technical defect was identified as a minor production issue. Nevertheless, the construction of an osmotic pump is much less complex than other lengthening mechanisms on the market (e.g. motorized or magnetic), the latter with a reported 32% device related complication rate [15]. Therefore, in comparison, the implant related failure rate should be less when distracting with the osmotic pump.

Further limitations are the comparability of the two *in vitro* setups because of the rest phase. In the first setup, four *in vitro* pumps were exposed to a temperature of 42 °C and three pumps were exposed to a temperature of 39 °C, each with an initial rest phase. In the second setup, in which the temperature dependence was further investigated, three pumps were started at 32.2 °C, at 38.6 °C, and at 46.3 °C without an initial rest phase.

Another limitation is that the *in vivo* pumps were not tested under load. *In vitro*, preliminary tests were performed with different static and dynamic axial forces. This showed that the starting time as well as the elongation per day is influenced by these forces. This must be taken into account whenever the osmotic effect is used.

In summary, the present study shows that elongation of an osmotic pump designed for osteodistraction is generally feasible under *in vivo* conditions. Eight out of eleven implants distracted successfully both *in vivo* and *in vitro* after an intended initial rest phase of four days.

Despite some limitations, the study showed that constant elongation of an osmotic driven pump was possible *in vivo* and *in vitro* and furthermore also highlights the importance of temperature dependency.
